# Implications of EMG channel count: enhancing pattern recognition online prosthetic testing

**DOI:** 10.3389/fresc.2024.1345364

**Published:** 2024-03-04

**Authors:** Ann M. Simon, Keira Newkirk, Laura A. Miller, Kristi L. Turner, Kevin Brenner, Michael Stephens, Levi J. Hargrove

**Affiliations:** ^1^ Center for Bionic Medicine, Shirley Ryan AbilityLab, Chicago, IL, United States; ^2^ Department of Physical Medicine and Rehabilitation, Northwestern University, Chicago, IL, United States; ^3^ Department of Biomedical Engineering, Northwestern University, Evanston, IL, United States

**Keywords:** below-elbow amputation, artificial hand, channel reduction, muscle signals, myoelectric control, outcome measures, surface electromyography

## Abstract

**Introduction:**

Myoelectric pattern recognition systems have shown promising control of upper limb powered prostheses and are now commercially available. These pattern recognition systems typically record from up to 8 muscle sites, whereas other control systems use two-site control. While previous offline studies have shown 8 or fewer sites to be optimal, real-time control was not evaluated.

**Methods:**

Six individuals with no limb absence and four individuals with a transradial amputation controlled a virtual upper limb prosthesis using pattern recognition control with 8 and 16 channels of EMG. Additionally, two of the individuals with a transradial amputation performed the Assessment for Capacity of Myoelectric Control (ACMC) with a multi-articulating hand and wrist prosthesis with the same channel count conditions.

**Results:**

Users had significant improvements in control when using 16 compared to 8 EMG channels including decreased classification error (*p* = 0.006), decreased completion time (*p* = 0.019), and increased path efficiency (*p* = 0.013) when controlling a virtual prosthesis. ACMC scores increased by more than three times the minimal detectable change from the 8 to the 16-channel condition.

**Discussion:**

The results of this study indicate that increasing EMG channel count beyond the clinical standard of 8 channels can benefit myoelectric pattern recognition users.

## 1 Introduction

Myoelectric pattern recognition control is a commercially available option for upper limb prosthesis control. Following transradial amputation, the most common major upper limb amputation (1), users can make physiologically appropriate muscle contractions of their phantom hand or wrist. Measurement via surface electromyography (EMG) of their residual forearm muscles and identification of these patterns of activity can be used to intuitively control similar prosthesis movements. For transradial users, the use of myoelectric pattern recognition, particularly after a period of home usage, has shown improvements over two-site agonist-antagonist control in some studies (2, 3), while other laboratory-based studies controlling fewer movements demonstrate similar outcomes between the two systems (4). Due to the amount of dexterity lost in the missing hand, there is still much room to improve users' control and functional capabilities with any of these prosthetic systems.

Multiple review articles cover decades of pattern recognition research aimed at increasing the accuracy of powered upper limb devices including investigating various features and classification techniques, arm position, electrode shift during don-doff cycles (i.e., the process of putting on and taking off a prosthetic device), and proportional control (5–7). The EMG measurement system is an important part of the upper limb prosthetic system, and often channel optimization or channel reduction investigations are a subsection of pattern recognition studies. In a study quantifying control of hand and wrist using 12 uniformly placed surface EMG channels, authors indicate that offline analyses show that six optimally chosen channels only reduced accuracies from 93.1% to 91.5% for 6 movements (8). When investigating the impact of arm position and integrating EMG with accelerometer data, researchers noted that for five individuals with a transradial amputation, offline classification error only minimally increased when the number of channels was reduced from eight to two (9). When classifying finger movements for transradial users, a subset of 6 EMG channels classified 12 different individual finger movements with 90% accuracy, which was very similar to the accuracy when using all 11 channels (10). Using the dataset from (10), independent component analysis and clustering methods were used to find a reduced set of four EMG sensors that did not reduce overall accuracy (11). Similar results classifying finger movements were observed using a genetic algorithm to reduce features and channels: data from one individual with a transradial amputation and five with no limb absence indicated that 8–11 of the 16 EMG channels recorded in the study could be eliminated without sacrificing classification accuracy (12). Our own prior work using offline analyses suggested that finding an optimal subset of 3 channels from a set of 16 channels does not provide statistically significant reduction in classification accuracy (13).

While these surface EMG channel reduction results are encouraging and could reduce complexity of the pattern recognition EMG socket, these results are from offline analyses. Offline analysis may not always have a strong correlation with online pattern recognition performance metrics (14, 15). Although high offline accuracy may be necessary, it alone may not confirm good functional real-time control of a pattern recognition prosthesis (15). Alternatively, real-time control of a virtual prosthesis may be used to enhance offline analyses. For example, virtual prosthesis control has been found to be predictive of functional performance with a physical prosthesis: control of a virtual prosthesis on the Target Achievement Control (TAC) Test (16) was correlated with control of a physical prosthesis during several clinical outcome measures including the ACMC, the Southampton Hand Assessment Procedure, and the Box and Blocks test (17). Additionally, channel reduction studies use pre-gelled silver/silver chloride electrodes as an EMG interface, whereas clinical interfaces usually involve dry stainless steel domes embedded in a socket. This difference may also affect results. Therefore, investigation of performance with a clinical interface and in real-time is necessary to expand insight on whether or not reducing the number of EMG channels truly affects control.

The three upper limb pattern recognition systems that earned Food and Drug Administration (FDA) class II clearance (18–20) currently record up to 8 EMG channels. At the time they first became available, this was an increase from two-site agonist/antagonist control. Maintaining good skin contact with all 8 bipolar EMG channels (up to 17 domes embedded in a socket) was initially a concern. The success of these commercial systems indicates that good well-fitting sockets with 8 channels can be achieved. Clinical practice of electrode placement involves muscle palpation and selecting sites that have underlying muscle that maintain good contact during use (21). Clinical selection of these 8 channel locations is likely different than the optimal reduced channel sets found in the literature. If space is limited due to residual limb length, electrode contact points can even be shared between EMG channels. If space is not limited, the impact of more contact points and more EMG channels embedded in a prosthetic socket is untested.

The effect of EMG channel count on real-time prosthesis control with users in the limb loss population has not been directly investigated. This study serves to fill that knowledge gap for below-elbow prosthesis control. Individuals with no limb absence and individuals with unilateral transradial amputation controlled a virtual prosthesis in real-time using pattern recognition configured with 8 and 16 channels of EMG. Individuals with transradial amputation additionally used a physical prosthesis to complete the ACMC under the same channel conditions. We hypothesized that increased EMG channel count would result in improved control of both the virtual and physical prostheses. If supported, users may gain functional benefits of increasing the number of EMG channels in clinically available pattern recognition systems.

## 2 Methods

### 2.1 Participants

Individuals with no limb absence and individuals with a transradial amputation between the ages of 18 and 95 were recruited at the Shirley Ryan AbilityLab in Chicago, IL, for this study. Inclusion criteria for the individuals with an amputation also included history of a unilateral upper limb amputation below the elbow, the ability to use a prosthesis under myoelectric control, and residual limb length large enough to accommodate 33 electrode contacts. Exclusion criteria included cognitive impairment, evaluated subjectively during the consenting process that would interfere with their understanding of study requirements or any significant comorbidity that would preclude completion of the study. The study was approved by the Northwestern Institutional Review Board (STU00216244 and STU00015912), and all participants provided written informed consent to participate.

### 2.2 EMG configuration and study prosthesis

For all participants, 33 electrodes were placed on the surface of the forearm in order to measure from 16 bipolar EMG channels with one reference (i.e., ground) electrode. For individuals with no limb absence, silver/silver chloride electrodes were placed with an approximate 3 cm inter-electrode distance in two circumferential bands: 8 pairs in a band at the proximal portion of the residual forearm around the apex of the muscle bulge and another 8 pairs in a band distal to the first (Table 1). All 16 EMG channels were recorded for all trials. For the 8-channel condition, all 8 channels of EMG in the proximal band were used to train to the pattern recognition system. EMG signals were amplified and digitized using a Texas Instruments ADS1299 chip sampled at 1,000 Hz.

**Table 1:** Protocol overview.

Participants	EMG setup	Virtual TAC Test	Physical ACMC and survey
No limb absence	• Silver/silver chloride pre-gelled electrodes • 3 cm inter-electrode distance • Two circumferential bands of electrodes: 8 pairs in proximal band and 8 pairs in distal band	*N* = 6	
Transradial amputation	• Custom socket fabrication • Stainless steel dome electrodes • 3–5 cm inter-electrode distance • Two circumferential bands of electrodes: 12 pairs in proximal band and 4 pairs in distal band	*N* = 4	*N* = 2

**Figure 1:**
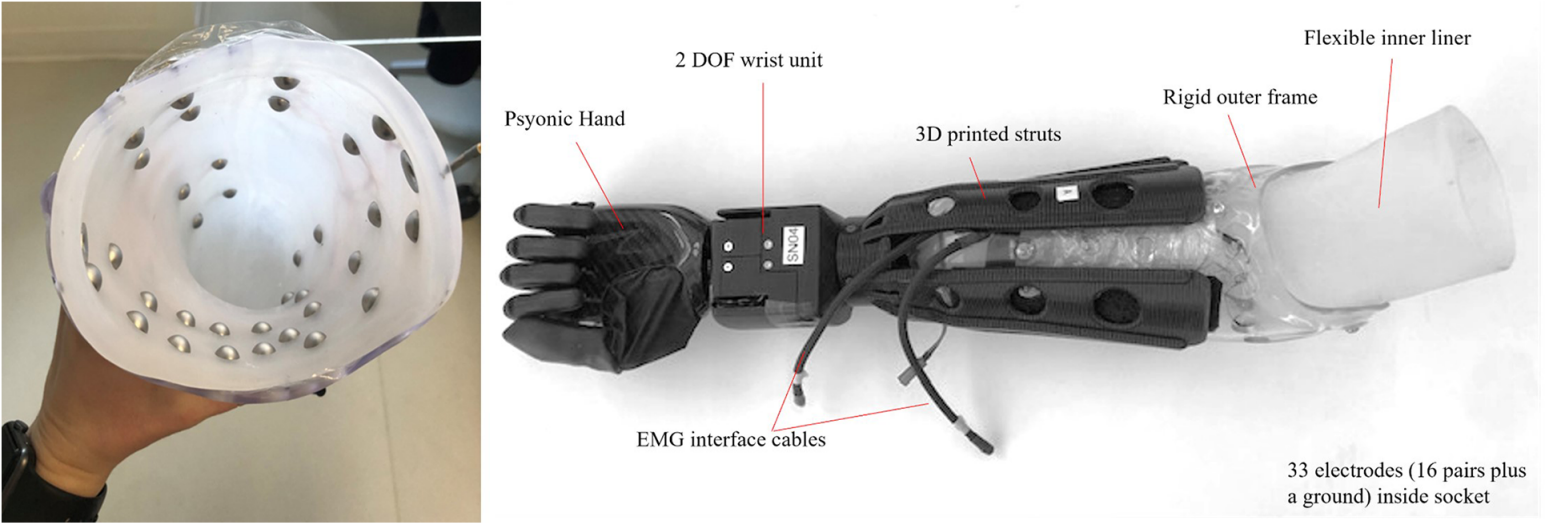
Physical prosthesis fabrication including (*left*) flexible inner liner with 33 electrode domes and (*right*) Psyonic Ability Hand and custom powered wrist.

For individuals with a transradial amputation, custom sockets were fabricated (Figure 1) for use during both the virtual and physical prosthesis testing. Existing, well-fitting sockets were duplicated and a new socket was fabricated with a flexible inner liner. A clinician palpated the residual limb according to clinical practice to determine the locations of the bulk of the forearm muscles. Two circumferential bands of electrodes with an approximate 3–5 cm inter-electrode distance, based on residual limb length, shape and scar tissue, were selected over the forearm muscles: a proximal band consisting of twelve electrode pairs and a distal band of four electrode pairs. All 16 EMG channels were recorded. For the 8-channel condition, six electrode pairs from the proximal band and two pairs from the distal band were used to train the pattern recognition system. Electrode locations were transferred to the flexible inner liner, and stainless steel dome electrodes were installed (Figure 1, *left*). The study prosthesis consisted of the flexible inner liner and rigid vivak frame with 3D printed connections to a custom two-degree-of-freedom wrist and the Psyonic Ability Hand (22) (Figure 1, *right*). This prosthesis was used for both virtual and physical prosthesis testing.

### 2.3 Channel count evaluation

#### 2.3.1 Virtual prosthesis testing

Participants with a transradial amputation wore the study prosthesis, but hand and wrist motors were turned off. The raw EMG measured from the flexible liner was sent to a desktop computer and displayed on a monitor to verify electrode contact and to control a virtual prosthesis displayed in front of the individual. The virtual (i.e., graphical) environment used in this study was non-immersive.

All participants trained a pattern recognition system to recognize hand and wrist movements using verbal and screen-guided prompts, making natural muscle contractions that mimicked a sequence of pictures on the monitor (23). All users were instructed to keep their forearm unsupported during calibration and training. Individuals with no limb absence trained hand open and close, wrist pronation and supination, wrist flexion and extension, and rest. Individuals with a transradial amputation trained hand open, hand close in chuck and key grip, wrist pronation and supination, and rest. During this seated calibration participants held each contraction for 3 s and repeated each motion four times. For individuals with a transradial amputation, training data collection was also prompted by a clinician, and one grip was trained first before adding the second grip. Additionally, for these individuals all virtual prosthesis use (calibration and testing) occurred with the physical prosthesis donned, forearm unsupported to create a weighted environment within the prosthetic socket, and hand/wrist motors turned off.

The pattern recognition classification system used was well established (24, 25) and similar to what is clinically available. EMG data from 8 or 16 channels were segmented into 200 ms analysis windows with a 25 ms update rate, four time domain and six auto-regressive features were extracted, and a linear discriminant analysis classifier (5, 26, 27) used to decode hand and wrist movements. Movement speed was proportional to EMG amplitude (28), and a decision-based velocity ramp was used to limit speed if movement decisions were not consistent (29).

**Figure 2:**
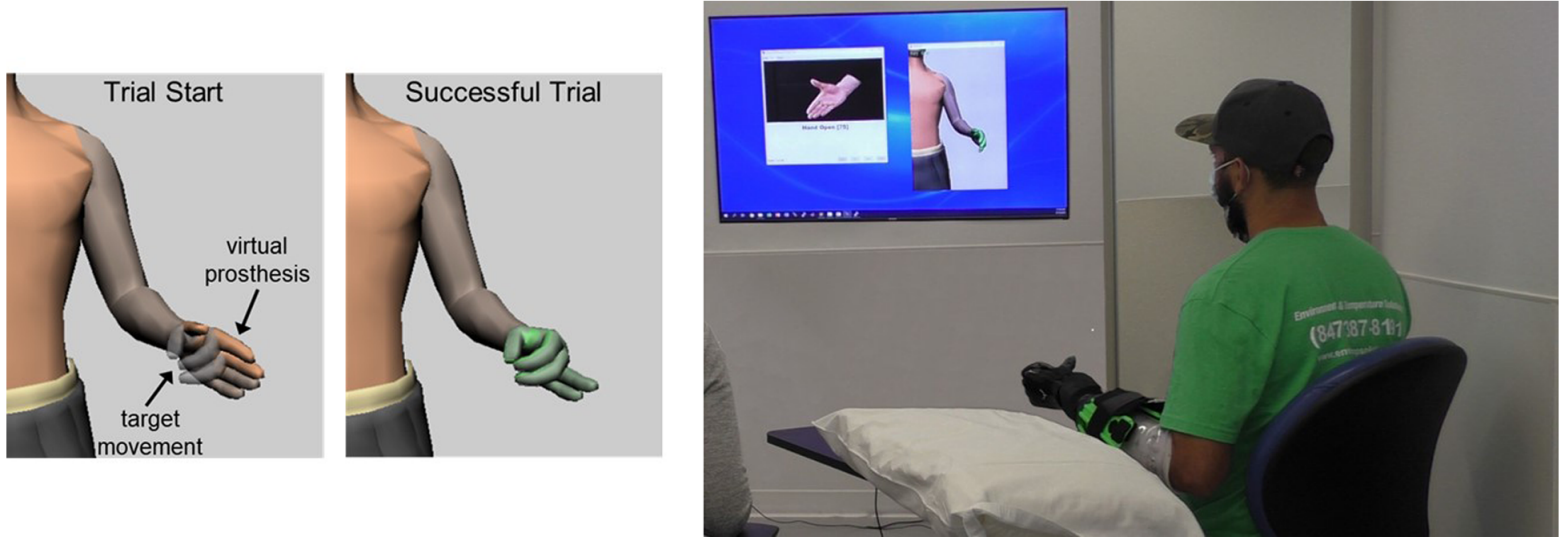
Target Achievement Control (TAC) Test. Participants moved a virtual prosthesis into target posture. The virtual hand turned green when target was reached within acceptable tolerances. Participants with transradial amputation wore the study prostheses during virtual testing. All users were instructed to keep their forearm unsupported during testing.

After calibration, participants had sequential control of the trained movements and controlled a virtual prosthesis in a real-time non-immersive virtual environment. EMG quality was monitored to promptly identify and resolve obvious sources of noise (all participants) or socket-fit issues (transradial participants). When multiple grips were calibrated, users had to fully open the virtual hand to switch grips. After they demonstrated control of the virtual prosthesis, they practiced repetitions of the Target Achievement Control (TAC) Test. During the TAC Test, participants are instructed to move the virtual prosthesis such that it matches a target posture (Figure 2) within an acceptable tolerance on each trained degree of freedom (±5 degrees for participants with no limb absence and ±10 degrees for participants with transradial amputation). All participants had at least one practice session prior to testing.

Data collection began with two screen-guided calibrations, which were used to train the virtual prosthesis. Similar to practice, each calibration had two repetitions of 3 s contractions for each movement and a rest period of 2 s between movements. After participants re-acclimated to controlling the virtual prosthesis, they performed six repetitions of each one-degree-of-freedom movement target in the TAC Test. Each condition (8 and 16 channels) was randomized as were the repetitions within each condition trial. Breaks were provided as necessary between TAC Test trials with a longer break between channel conditions. A single researcher set the channel condition during testing. The participant and other individuals in the room (prosthetist, occupational therapist, and additional researchers) were blinded to channel condition throughout the testing. At the end of the session, four more repetitions of each motion were collected via screen-guided calibration for offline analysis.

After each condition, participants in the transradial group completed a questionnaire aimed at assessing the user's perceived control, which asked them to rank on a scale from 1 (very easy) to 5 (very hard) how difficult it was to move the prosthesis in each of the five movements (wrist supination, wrist pronation, hand open, chuck grip, and key grip). A total score of 5 indicated that all movements were easy to achieve and, a total score of 25 indicated all movements were very hard.

TAC Test performance metrics were averaged across all movements. Metrics included: failure rate (the percentage of trials participants failed to achieve the target posture within 20 s), completion time (for successful trials, the time to achieve the target posture), and path efficiency (the shortest path to the target posture divided by the total distance traveled during the trial). Additionally, the two reserved calibrations collected at the end of the experiment were used to calculate offline classification error. Since some users had delayed onset or early termination of their muscle contractions, classification error was calculated as a false activation rate: muscle contraction movements that were incorrectly classified as rest were not included as errors.

#### 2.3.2 Physical prosthesis testing

In an additional testing session on a separate day, individuals with a transradial amputation used the study prosthesis with the 8- and 16-channel conditions. The Psyonic Ability Hand had the capability to close in multiple grips, but only key and chuck grip were calibrated for this preliminary testing. Additional custom post-processing was necessary to ease how the hand physically changes grips because the Psyonic Hand does not need to be fully open to change grip patterns. Pilot testing indicated that if grip decisions were not consistent, the hand would change to a different grip even if an object was being held. Previous approaches to resolve this issue include requiring the user to fully open the hand and/or perform a hand open signal for a set time duration before allowing the system to change grips (30). In this study, users were required to perform a strong hand open signal for a set duration prior to the hand changing grips. Clinician and user feedback was used to set the strength and duration thresholds of the hand open contraction for each user.

Like the virtual session, participants donned the study prosthesis and calibrated the same set of movements. After the device was calibrated, the hand and wrist motors were turned on to allow the participant to practice using the prosthesis. Practice involved working with an occupational therapist to control each motion of the prosthesis, stacking blocks, folding a towel, and other tasks in both a seated and standing position.

A single researcher set the channel condition in a randomized order so all other individuals, including the user, were blind to the channel condition. After the channel condition was set, participants practiced with the prosthesis for an additional 5 min. Re-calibration was allowed based on the clinical discretion of the blinded occupational therapist/prosthetist. Participants performed a single trial of the ACMC with each channel condition. The ACMC is an observational assessment measuring the user's quality of prosthetic hand movements during a two-handed functional task (31, 32). The ACMC outcome was videotaped while users packed luggage, gathering items from various size containers and locations, packing and folding them into a suitcase. After each channel condition, participants completed the same questionnaire aimed at assessing their perceived control. If the participant recalibrated during the previous condition, the system was reverted to the original calibration data prior to starting the 5-min practice with the other channel condition (again with the opportunity to recalibrate), thus establishing a standard baseline of control for each condition. At the end of the session, four more repetitions of each motion were recorded via screen-guided calibration for offline analysis. A certified occupational therapist who was also blinded to the channel condition scored the assessment using the video.

#### 2.3.3 Statistical analysis

Analysis of variance statistical analyses were conducted utilizing Minitab Statistical Processing Software (Version 21) to assess the impact of EMG channel count on various measures associated with the TAC Test, including classification error rate, failure rate, completion time, and path efficiency. To accommodate the hierarchical structure of our data, where multiple measurements were taken from each participants, linear mixed effects models were employed. Participant was incorporated as a random effect to account for inter-subject variability, while population (categorized into no limb absence or transradial amputation) and channel count (8 or 16) were included as fixed effects to evaluate their influence on the dependent variables.

For each dependent variable, a separate linear mixed effects model was specified. The model fitting process involved the estimation of fixed effects to understand the relationship between the dependent variables and our predictors, while random effects were used to model the variability attributable to differences across participants. The significance of fixed effects was determined using likelihood ratio tests, comparing the full model containing the predictors with a reduced model excluding the effect in question. This approach allowed for the assessment of whether EMG channel count, as well as the population category, significantly affected the outcomes of interest.

## 3 Results

### 3.1 Participants

Ten individuals (six with no limb absence and four with a unilateral transradial amputation) participated in this study (Table 1). The no limb absence group consisted of 3 males and 3 females, were all right-handed, and all used their right arm to control the virtual prosthesis. The transradial group was all male, and all reported limb loss secondary to trauma. Additional demographics for participants with transradial amputation are listed in Table 2.

**Table 2:** Demographics of individuals with a transradial amputation.

ID	Age	Years since amputation	Prosthesis side	Previous handedness	Residual limb length	TMR	PR experience	Recent home prosthesis use
S1	36	9	Left	Right	Long (300 mm)	Y	Y	Non-user
S2	60	44	Right	Right	Medium (210 mm)	N	Y	Myoelectric (2-site), 8+ h/day, 7 days/week
S3	29	3	Left	Right	Medium (245 mm)	N	Y	Myoelectric (2-site), 4-8 h/day, 5-7 days/week
S4	34	6	Left	Right	Medium (225 mm)	Y	Y	Myoelectric (PR) or BP, <4 h/day, 1–7 days/week

TMR, targeted muscle reinnervation (24); PR, pattern recognition; 2-site, two-site agonist-antagonist control; BP, body-powered prosthesis.

### 3.2 Virtual prosthesis results

**Figure 3:**
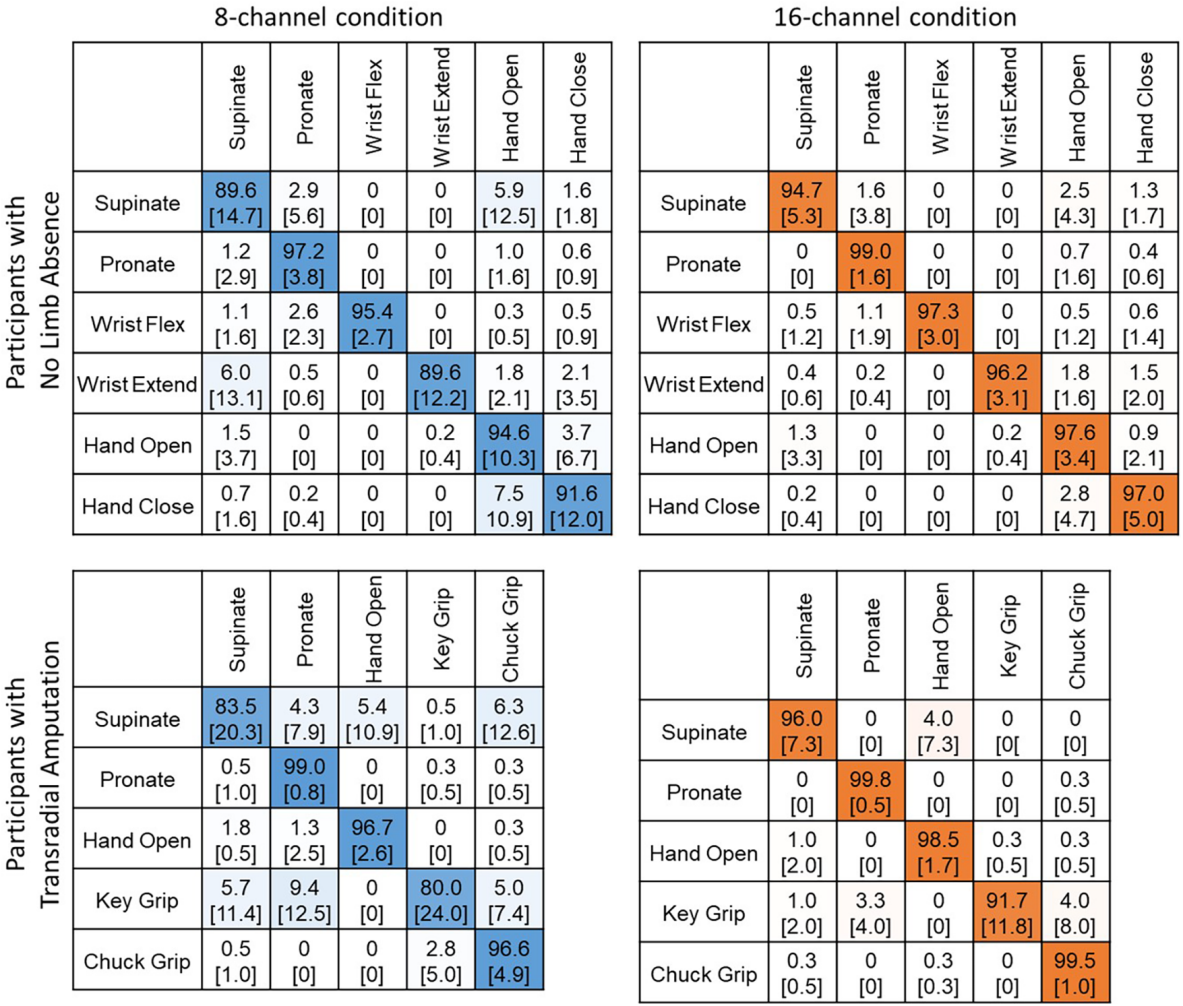
Confusion matrices show improvements in movement prediction for the 16-channel system compared to the 8-channel system for both participants with no limb absence (*top*) and transradial amputation (*bottom*).

All participants successfully completed the virtual environment testing. Offline classification error significantly decreased for the 16-channel system compared to the 8-channel system (*p* = 0.006). There were no differences between population (*p* = 0.785). For users with the no limb absence, false activation error rates for six movements were 7.0% [6.1, S.D.] for 8 channels and 3.0% [2.6] for 16 channels. For users with transradial amputation, false activation error rates for five movements were 8.8% [7.5] for 8 channels and 2.9% [4.1] for 16 channels. Confusion matrices for these conditions are displayed in Figure 3.

**Figure 4:**
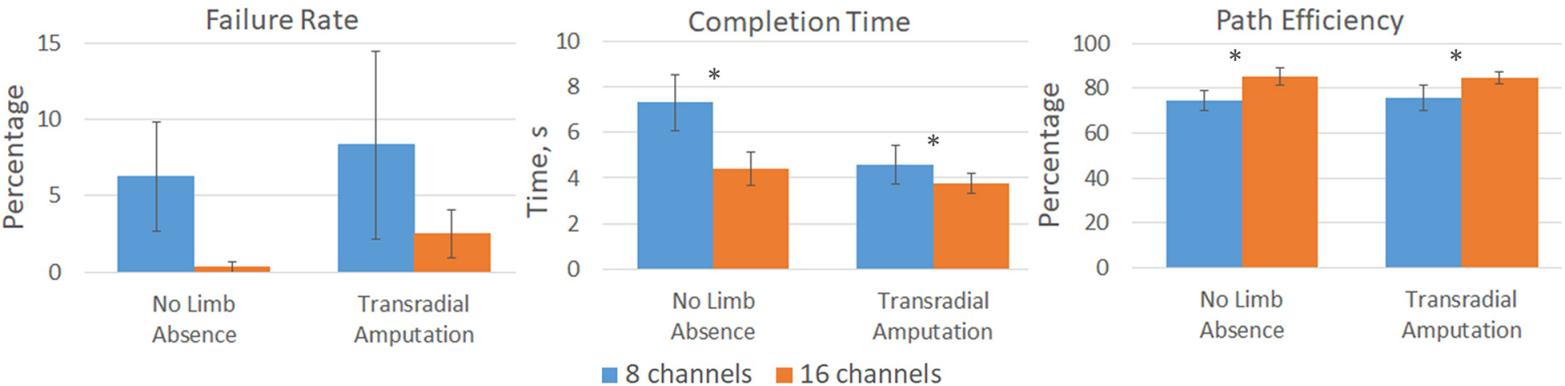
Virtual prosthesis TAC Test results for participants with no limb absence and with transradial amputation for both the 8-channel (*blue*) and 16-channel (*orange*) systems. Significant improvements, denoted by *, in completion time (p = 0.019) and path efficiency (p = 0.013) were measured for the 16-channel system compared to the 8-channel system. Failure rate was not statistically significant between channel count (p = 0.082).

TAC Test metrics for both groups showed an overall improvement in control for the 16-channel count condition compared to the 8-channel count (Figure 4). When using 16 channels compared to 8 channels, average failure rates decreased from 6.2% [8.8] to 0.3% [0.8] for the group with no limb absence and from 8.3% [12.3] to 2.5% [3.2] for the group with transradial amputation. However, these changes were not statistically significant between channel count (*p* = 0.082) or population (*p* = 0.525). Completion times decreased from 7.3 s [2.9] to 4.4 s [1.8] and 4.6 s [1.7] to 3.7 s [0.9]. These changes were statistically significant for channel count (*p* = 0.019) but not population (*p* = 0.962). Path efficiency increased from 74.8% [10.9] to 85.1% [9.3] and 75.9% [11.3] to 84.5% [5.3] for the groups with no limb absence and transradial amputation, respectively. These changes were statistically significant for channel count (*p* = 0.013) but not for population (*p* = .962).

### 3.3 Physical prosthesis results

Two individuals with a transradial amputation (S1 and S4) successfully completed the physical prosthesis testing. The other two participants' (S2 and S3) residual limb became quite fatigued when practicing using the study prosthesis, likely due to its length and weight. Since they were at elevated risk for fatigue and proximal joint discomfort, they did not attempt the ACMC.

**Figure 5:**
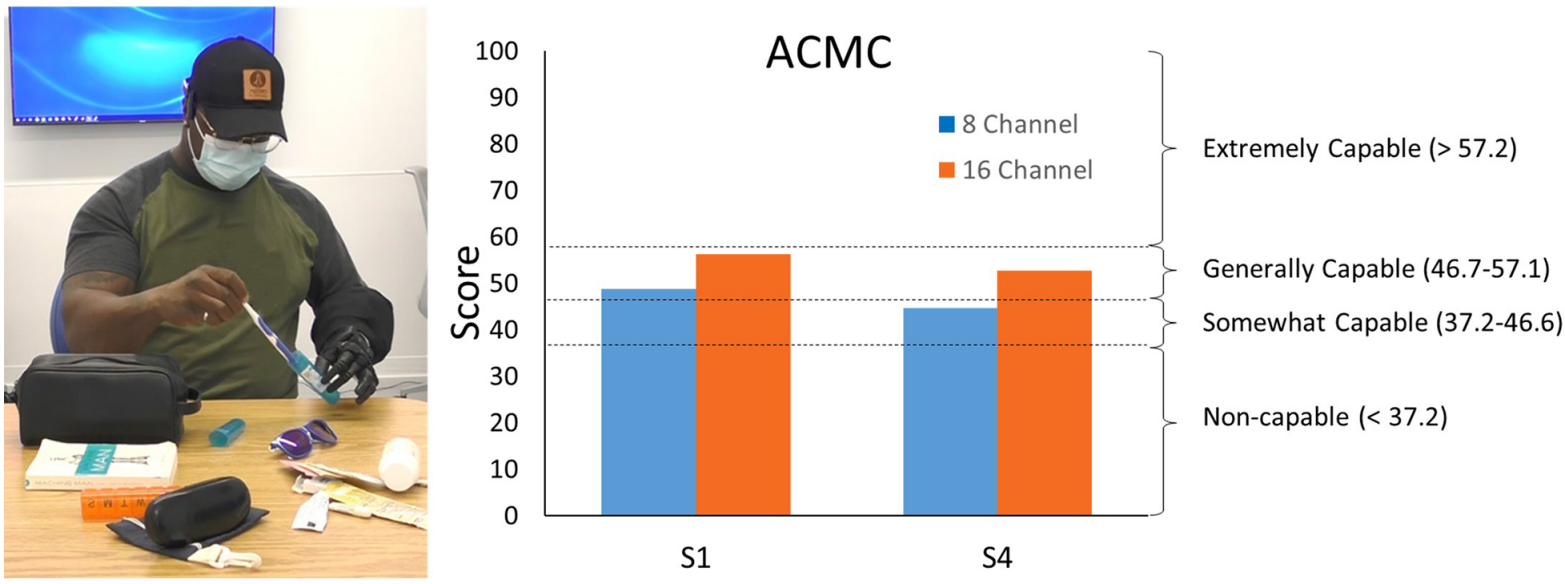
Individual with a transradial amputation packing luggage items (*left*) as a part of the ACMC. ACMC scores demonstrate increased capabilities with the 16-channel system compared to the 8-channel system (*right*).

Participants S1 and S4 demonstrated increased capabilities with the 16-channel system compared to the 8-channel system (Figure 5). S1 score increased from 48.7 to 56.3 and S4 increased from 44.6 to 52.8. Offline false activation rate error slightly decreased for the 16-channel system, 10.0% [2.4], compared to the 8-channel system, 12.8% [4.2].

### 3.4 Transradial user questionnaire results

**Figure 6:**
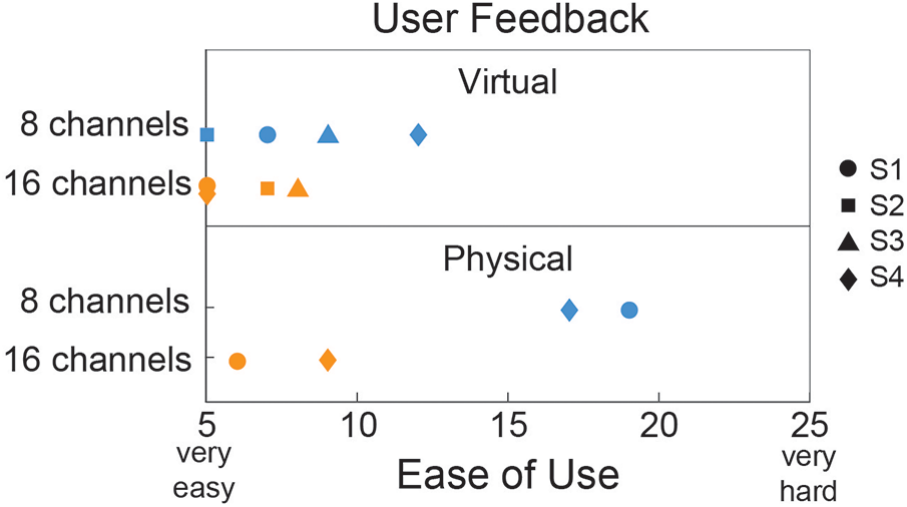
Ease of use questionnaire given to individuals with transradial amputation for both the virtual (*N* = 4) and physical (*N* = 2) prosthesis testing. A total score of 5 indicated that all motions were “very easy” and a total score of 25 indicated that all motions were “very hard”.

Since individuals with an amputation were the target population, they were the only group surveyed on their perception of control between the two different channel conditions. They were wearing and subjected to the weight of the study prosthesis for both the virtual and physical prosthesis testing but only interacting with the device for the physical testing. Figure 6 shows that, on average for the virtual testing, users perceived easier control with the 16-channel system compared to the 8-channel. A similar trend was seen for the physical prosthesis but with a much larger gap in ease of use between the two different channel conditions.

## 4 Discussion

To the authors' knowledge, this is the first study that evaluates EMG channel count during real-time pattern recognition control with end users. Results support our hypothesis that real-time control with a pattern recognition system configured with 16 EMG channels provides better control than one configured with 8 EMG channels. Both groups of participants (individuals with no limb absence and with unilateral transradial amputation) were able to complete TAC Test trials in significantly less time with significantly increased path efficiency with the 16-channel system. Participants with a transradial amputation also demonstrated increased capabilities while using a physical prosthesis to perform an outcome measure with the 16-channel system. Furthermore, survey results from transradial users indicate that they can perceive this control improvement in both the virtual and physical environment.

Physical prosthesis testing showed improvements in the ACMC score when using the system with more EMG channels: both participants' scores increased (S1 by 7.6 points and S4 by 8.2 points). This increase in ACMC score is more than three times the minimal detectable change of 2.5 for the ACMC scored by a single rater. This outcome measure demonstrates that these improvements in control are clinically relevant as one user's scores indicated a move from a category of “somewhat capable” to “generally capable”. Survey results also indicate an expected shift in decreased ease of use from the virtual to the physical environment. Physical prosthesis control is more difficult because weight, limb position, and fatigue likely have a larger impact. Notably, EMG channel count had a much larger impact on ease of use with a physical device compared to a virtual prosthesis. S2 and S3’s inability to complete physical prosthesis testing likely is a result of the selection of physical device. Despite participants S2 and S3 having residual limb lengths similar to S4, during use the device weight and length led to substantial muscle fatigue for them, making it impractical for use under either channel condition. An alternative study device or device configuration (e.g., single degree-of-freedom hand with two degree-of-freedom wrist) might have led to reduced device weight or length or different weight distribution, potentially enabling these participants to complete the channel condition study with a physical prosthesis.

False activation rates in this study were within the ranges of offline errors previously published for transradial users (i.e., classification accuracy converted to error rate). Error rate for the 8-channel condition was 8.8% for five movements (i.e., two wrist movements, hand open, and two grips): Li et al. found six optimally placed EMG channels resulted in an 8.5% error rate for six movements (four wrist movements, hand open, and hand close) (8), and Geng et al. (9) found 7.3% error for 6 intra-limb movements (four wrist movements, hand open and hand close). Error rates measured in this study, 2.9% with 16 EMG channels, are lower than these previously published results.

This work investigated channel count as it relates to an input into pattern recognition systems. However, there are other alternative methods of EMG prosthetic control. For example, multi-channel EMG signals can be decomposed to identify individual motor unit activity (33). This type of approach uses source separation techniques and often requires higher channel counts than typical pattern recognition systems. When successfully implemented, this approach has been used with individuals with no limb absence and two with limb difference (one transradial congenital absence and one with transradial amputation) to decode six wrist motions, showing hat on average 16 ±7 motor units were identified per motion with greater than 85% accuracy (34) and can occur in real-time (35). For individuals with no limb absence, dimensionality-reduction using a nonlinear autoencoder has shown promise for control of a high-dimensional virtual hand with only four EMG signals (36). It is possible that incorporating more channels into such a system may further improve performance, perhaps at the expense of a more extensive training data set for configuration of the autoencoder.

Virtual performance metric trends for the TAC Test were similar between groups even though the systems between groups were not the same. While the electrodes used for each population were different (i.e., pre-gelled silver/silver chloride for the group with no limb absence and dry stainless steel domes for the group with a transradial amputation) they both represent common electrode types used in upper limb pattern recognition research. Results are promising in that the trend towards improved control with 16 EMG channels was independent of these electrode differences. Stainless steel dome electrodes (i.e., the clinical interface for EMG controlled transradial prostheses) have higher impedance, poorer skin/electrode impedance matching and more electrode liftoff compared to the pre-gelled silver/silver chloride self-adhesive electrodes. Channel arrangement was slightly different: the 8-channel condition for individuals with no limb absence included only the proximal ring of electrodes whereas the 8-channel condition for individuals with transradial amputation included six channels in the proximal ring and two in the distal ring. Both of these EMG configurations are reasonable choices that potentially could be clinically implemented and both lead to similar improvements for the 16- vs. 8-channel conditions for completion time and path efficiency. They are, however, different than channel reduction studies that often reduce the number of channels to an optimal set of four to eight found via a search algorithm. In this way, the 8-channel condition may be slightly underestimating control performance of an optimal 8-channel system. But, this may be more clinically relevant since, currently, there is no quantitative mechanism to select optimal channels for individual users in the clinic. Another difference involves the trained and tested movements: the no limb absence group controlled a virtual two degree-of-freedom wrist and a one degree-of-freedom hand, whereas the transradial amputation group controlled a virtual one degree-of-freedom wrist and hand with two grips. The TAC Test was programmed to be slightly more difficult for the no limb absence group (i.e., smaller window of acceptable tolerances of all degrees-of-freedom). These variations were included to test differences more broadly in channel count.

A clinically-relevant choice during testing was to use the same EMG interface during virtual prosthesis testing that individuals with a transradial amputation would use during physical prosthesis testing. These participants wore and supported the weight of the study prosthesis during virtual testing. While a custom socket is not always available for real-time virtual testing, when available, it does provide a more real-world environment for measuring EMG. Additionally, it was important to confirm that 33 dome electrodes could be installed into an upper limb socket. It was noteworthy that inclusion in this study required having a residual limb length large enough to accommodate 33 domes; therefore there is a subset of users in which there is not enough room. These results, however, would indicate from a merely channel count perspective, to include more than 8 and up to 16 channels if possible.

Clinically, these results may be challenging to implement as it doubles the complexity of the EMG-socket interface. Maintaining good electrode contact during home use is difficult (30); EMG channels are susceptible to signal noise. While the reliability of surface EMG recordings over time may be challenging, research into automatic noise detection and fault-tolerant systems is showing promise to allow users to maintain reliable control even if and when EMG signal noise occurs (37, 38). This is true regardless of the control strategy employed.

This study had some limitations including the low number of participants with transradial amputation and limitation on running statistics. The protocol involved making a socket with embedded electrodes for each participant and having the participant wear the prosthesis for both the virtual and physical testing. For two participants, the prosthesis weight and length was too much for them to support during physical prosthesis use. It is possible that if either the custom wrist or the Psyonic Ability hand was swapped out for a shorter or lighter version, they may have been able to complete the ACMC. Another limitation to this study of channel count was that we only included participants who had enough room in their socket for 33 EMG domes. Although untested in this study, it would be valuable to know if utilizing electrode contact sharing to achieve 16 EMG channels for users with shorter residual limbs results in similar control improvements above the now standard 8 EMG channels.

## 5 Conclusion

Contrary to the standard 8 EMG channels currently used for commercial upper limb pattern recognition systems, our results indicate that increasing the number of EMG channels can lead to improvements in both offline and online control. Our work does not imply that existing control systems work poorly, merely that more capable systems could be created in the future. Importantly, these results are consistent for individuals with transradial amputation during both virtual and physical prosthesis testing. Improvements were not only perceptible to the end users but also measurable by means of the TAC Test and ACMC.

## Data Availability

The raw data supporting the conclusions of this article will be made available by the authors, without undue reservation.
